# Dietary Restriction in *Drosophila:* Delayed Aging or Experimental Artefact?

**DOI:** 10.1371/journal.pgen.0030057

**Published:** 2007-04-27

**Authors:** Matthew D. W Piper, Linda Partridge

**Affiliations:** Johns Hopkins University School of Medicine, United States of America

## Abstract

Lifespan can be extended by reduction of dietary intake. This practice is referred to as dietary restriction (DR), and extension of lifespan by DR is evolutionarily conserved in taxonomically diverse organisms including yeast, invertebrates, and mammals. Although these two often-stated facts carry the implication that the mechanisms of DR are also evolutionarily conserved, extension of lifespan could be a case of evolutionary convergence, with different underlying mechanisms in different taxa. Furthermore, extension of lifespan by different methods of DR in the same organism may operate through different mechanisms. These topics remain unresolved because of the very fact that the mechanisms of DR are unknown. Given these uncertainties, it is essential that work on the mechanisms of DR is not clouded by imprecise descriptions of methods or by technical problems. Here we review the recent literature on DR in *Drosophila* to point out some methodological issues that can obscure mechanistic interpretations. We also indicate some experiments that could be performed to determine if DR in *Drosophila* operates through similar mechanisms to the process in rodents.

## Introduction

At the beginning of the 20th century, the first experiments studying the effects of environmental interventions on lifespan were undertaken using the fruit fly Drosophila melanogaster [1]. Of particular note is the work of Loeb and Northrop [2], which found that *Drosophila* maintained at lower temperatures were longer lived. Since metabolic rate in poikilotherms is determined by ambient temperature, it was proposed by Raymond Pearl that organisms have a genetically predetermined amount of energy to expend in a lifetime and, therefore, that the rate of its expenditure would determine lifespan [3,4]. The central mechanism of this “rate of living” hypothesis is that aging and subsequent death are brought about by an accumulation of the detrimental effects of the products of normal metabolism that cannot be adequately cleared.

At about the same time, Stefan Kopeć [5], working on the causes of death by starvation, reached the conclusion that it was brought about by “autointoxication” from products of hunger metabolism. In addition, he proposed that starvation provided “antitoxins” against those detrimental effects of normal metabolism referred to by Pearl above and, likewise, that normal feeding provided “antitoxins” against starvation. He thus reasoned that balancing these effects might benefit the organism and lead to prolonged lifespan. He tested this hypothesis experimentally in 1928 using *Drosophila* maintained on a regime of alternating periods of feeding and fasting [5]. Interestingly, Kopeć reported that the “…results pointed undoubtedly to the conclusion that periods of intermittent starvation for six hours out of every 24 increases longevity of the flies . . . ” [5]. However, examination of the data in the paper shows the effect was less than convincing, ranging from 16% shortening of mean lifespan to 17% extension, with an average effect over ten trials of 2% extension.

Seven years later, Clive McCay reported the effects of an intermittent feeding regime on the lifespan of white rats [6]. This resulted in males achieving almost double the lifespan of those fed *ad libitum,* an effect that has since been repeated in rodents many times using a variety of different methods, and now termed caloric restriction or dietary restriction (DR) [7]. We use the more general term “DR” in this review, because it covers both cases where total caloric intake correlated with extended lifespan and those where specific nutrients are of importance, leaving open which of these mechanisms is operative. Despite the initial lack of success that Kopeć [5] in fact had with the intermittent feeding DR protocol in flies, it has since been shown that it is possible, with appropriate techniques, to extend the lifespan of many organisms, including *Drosophila*, by reducing food intake (see Table S1 for a summary of DR experiments with flies and the range of techniques implemented). This has opened the way for the use of short-lived, invertebrate model organisms such as yeast, nematodes, and flies to investigate the mechanisms of DR that could also be operative in mammals.

A potential problem of comparative DR studies using distantly related species is that the mechanisms underlying the prolongation of life may not be conserved between different lineages during evolution, because the mechanisms by which DR extends lifespan within each species are unknown. To make progress with resolving the issue of evolutionary conservation, it is essential that studies of DR are reported in sufficient detail for the procedures to be replicated and that any intervention described as “DR” does in fact operate through a reduction in the supply of nutrients, rather than through the removal of some other detrimental effect. Despite the simplicity of this point, it is overlooked surprisingly frequently. Here, we consider how such technical issues could have affected conclusions of studies of DR in *Drosophila*. Resolving these issues are of central importance for uncovering the mechanisms of DR through comparative model organism studies.

## Restricting Access to Nutrients

The most practical method for implementing DR in *Drosophila* is by dilution of the food medium to which the flies have *ad libitum* access [8]. In addition to the work by Kopeć [5] cited above, several other laboratories have unsuccessfully attempted to implement DR in a variety of fly species using intermittent feeding regimes [9–12] (Table S1). This lack of success has been interpreted by some to indicate that DR does not work in flies [9,12,13]. An alternative explanation is that there is a detrimental effect of daily periods of extended starvation for flies that is not found for rodents when fed intermittently. This detrimental effect could counter-act any beneficial effects of underfeeding and so fail to extend lifespan. This could occur if the intermittently fed animals have specific nutritional needs during the starvation period that must be met for lifespan to be extended by intermittent feeding. Interestingly, Partridge et al. [14] found that when they intermittently fed yeast to flies (every sixth day) that had constant access to sugar-water, lifespan was extended by some 30% compared with flies fed yeast and sugar *ad libitum*. This contrasts with the negative results from other intermittent feeding studies, where the flies had constant access to water that did not contain sucrose [5,12]. These data indicate that a carbohydrate-enriched diet may be necessary to rescue a detrimental effect of starvation periods for *Drosophila*. The result also points to the specific nutritional effects of yeast being important for lifespan-extension by DR in flies, which has recently been confirmed [15].

## Food Dilution—Toxicity versus Nutrient-Dependent Lifespan Extension

**Figure 1:**
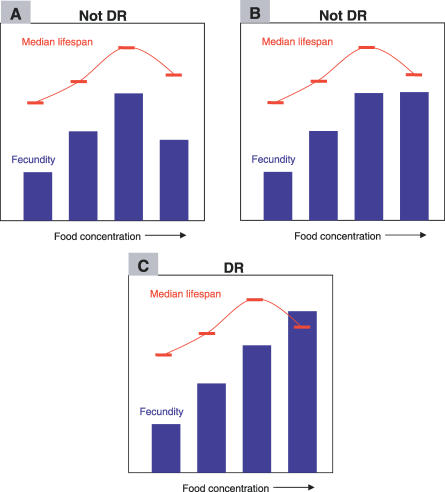
The Responses of Lifespan and Fecundity to Food Concentration That Is Required for DR Studies in *Drosophila* As food concentration increases from starvation, lifespan should increase to a peak at DR, from which it declines due to a nutrient-dependent effect of “high” food. If fecundity decreases (A) or is unchanged (B) by the nutrient increase that decreases lifespan, the most likely explanation for shortened lifespan is toxicity. To minimise the possibility that food toxicity is the explanation for the lifespan shortening at high food concentrations, it is important that daily and lifetime fecundity increase for the increases in food concentrations that decrease lifespan (C).

As mentioned above, DR in flies can be achieved by diluting the concentration of nutrients in their food medium, which is always present in excess (Table S1). One food type for *Drosophila* consists of an agar-gelled diet of dried autolysed yeast and sucrose [16,17]. The effect of changing the concentrations of these nutrients ranges from outright starvation at the lowest food levels, through a lifespan peak (DR), to a decrease in lifespan as nutrient concentration becomes “high” (Figure 1). The increase in lifespan seen from “high” levels of nutrition to DR is generally interpreted as a consequence of a positive effect of withdrawal of nutrients on the systems that ensure longer life, perhaps via a reduction in activities such as reproduction that may cause somatic damage [18]. A risk of using food dilution to implement DR is the possibility that flies compensate for lowered nutrient levels by increasing their feeding behaviour. At this stage, both evidence for [19] and against [15,20] compensatory feeding has been published. This discrepancy needs to be addressed with further work and may simply be due to the technical difficulties in determining the feeding rate of such a small organism (see Video S1 for an example of *Drosophila* feeding). It is estimated that *Drosophila* only consume between one and two microlitres of food per 24 hour period [19,21], 40-fold less than a blowfly for the same period of time [22]. Despite these difficulties, it is clear that egg-laying output, which is known to be under nutrient control [23–25], increases as food concentration increases [17,26]. Thus, if compensatory feeding does occur, it is not sufficient to overcome the degree of nutrient dilution in the diet, and the lifespan increase seen under DR correlates with a decrease in acquisition of biologically useful nutrients. However, this correlation does not reveal causation and an alternative explanation is that reduced food supply simply relieves “high”-food flies from a non-nutritional, toxic effect of the diet.

Dietary toxicity could occur from either the presence of a poisonous element in the food, or via an indirect effect of the food being nutritionally inadequate or unbalanced, or physically dangerous, or a source of infection. Whatever the precise mechanism, if toxicity increases as the food concentration increases, the effect is indistinguishable from DR, since lifespan is shortened as food concentration increases. For *Drosophila,* where the food is the nutrient and water supply as well as a large part of the physical environment, increasing the nutritional value of the food also results in an increase in the ratio of dissolved nutrients to water. This could therefore cause flies to suffer shortened lifespan due to increased food hardness or water stress. We have recently tested this possibility and shown that when experiments are carefully performed, neither food hardness nor water shortage account for the life-shortening effects of high-nutrient food [27]. It has also been suggested that increased nutrient content may enhance microbial growth, which could shorten fly lifespan by making the food more sticky or by encouraging a greater chance of lethal infection. Where these two factors have been tested, they have been shown not to affect lifespan [15,26]. For C. elegans, recent work has indicated that its commonly used laboratory feedstock, E. coli, has a toxic effect that shortens worm lifespan. In addition to worms surviving longer on killed *E. coli* media or in its complete absence [28–31], when worms were fed the soil bacterium B. subtilis instead of E. coli they were much longer lived [32,33]. This means that studies of “DR” in worms that rely on dilution of an E. coli food supply report the combined effects of reduced toxicity as well as reduced nutrient supply. In a more general sense, any natural food supply could contain toxins as well as nutrients, and therefore it is important to establish that any “DR” effect is in fact due to reduced intake of nutrients and not any direct or indirect toxicity effect of the food.

For flies, the problem of food toxicity is to some extent countered by using female egg-laying as a biologically relevant read-out of nutritional quality. If egg-laying increases with food supply, then it is reasonable to deduce that nutrient intake is increased. In combination with lifespan, egg-laying can indicate if food toxicity might be the cause of lifespan shortening, the argument being that if a fly does not increase its egg-laying for nutrient level increases that decrease lifespan, the food may be having a general toxic effect. For DR therefore, each increase in nutrient concentration that leads to a reduction in lifespan should be accompanied by an elevation in daily and lifetime fecundity (Figure 1). At the very least, this ensures that dietary types that are used for DR do result in increased nutrition over the range tested. A further test for food toxicity could be made using behaviour assays such as negative geotaxis [34] on young flies. Since the quality of industrially produced yeasts is dependent on the production method and seasonal quality of the feedstock, it is important that laboratories empirically determine whether they are working with yeast that is not toxic to flies. Unfortunately, not all studies have taken this precaution (Table S1). For instance, some work on the effects of dietary lipids on lifespan was performed without any simultaneous measure of egg laying or activity, thus making it impossible to know if increased food supply was in fact associated with increased nutrition, or if the short lifespans associated with elevated lipid supply were due to a nutritional effect or instead due to toxicity of the lipid sources added [35–37]. In some cases, such as experiments with males or sterile females, egg laying cannot be used to indicate the food quality. In these instances, informed choices can be made using data from fertile females of the same genetic stock or, as mentioned above, by the use of behavioural assays such as climbing ability. A biological indicator of nutrient quality other than lifespan is essential, because inter-species comparisons of the mechanisms of DR rely on the fact that DR is actually being studied in each organism.

One important line of work to uncover the mechanisms of DR has focussed on isolating the specific nutritional requirements for the effect on lifespan. The community working on DR in rodents has generally taken the view that reduced calorie intake is critical for the lifespan-extension associated with food reduction and has therefore adopted the term caloric restriction [7,38]. However, it is also acknowledged that other types of food restriction that do not affect calorie intake can also extend lifespan [7,39–46]. Each of these interventions may or may not lead to lifespan-extension through the same mechanisms. For instance, dietary restriction for a particular nutrient such as methionine [42,45] may in fact affect the utilization of other dietary components such that the organism ultimately experiences physiological energy restriction. Studies of the effect of exposure to diets of varying composition on food intake, and of the effects diet composition has on absorption of specific nutrients are needed to resolve these issues. In addition to these effects, there is evidence in worms and flies that olfaction of food, as well as food intake, can shorten lifespan [47,48]. One clear fact to emerge from the body of work on rodents is that, in addition to the data implicating reduced calorie intake as sufficient for lifespan extension, changes to either the quality or quantity of the protein component of the diet has the capacity to alter lifespan, even when caloric intake is equivalent between cohorts [39,40,44,45,49]. Recently, work on *Drosophila* has reached a similar conclusion, by showing that specific reduction of the yeast component in an otherwise isocaloric diet can extend lifespan to a similar degree as whole-food (yeast and sucrose) reduction, which also has lower caloric content [15]. Even more recently, an attempt has been made to use semi-defined media to study DR in flies [50]. This paper concluded that the protein component of the diet was critical for the effect. However, the flies on the semi-defined diet had dramatically reduced daily and lifetime fecundity and were not longer-lived than fully fed controls, indicating a toxic effect of the protein source (casein) being used. Thus it is important that future work focuses on developing appropriate semi- and fully-defined diets that avoid complications from toxicity.

An attractive candidate mechanism for the prolongation of life in response to food reduction, and more specifically protein shortage, is the activation of autophagy [51–53]. This process turns over subcellular material in response to low nutrients and is as evolutionarily conserved as the DR effect itself [54]. Autophagy is activated by reduced insulin and TOR (Target Of Rapamycin) signaling and is therefore enhanced when both the energy and amino acid status of the organism are lowered [55–57]. Autophagy acts to recycle damaged/malfunctioning proteins and organelles from the cell and may, therefore, as a by-product of this action, clear out damaged macromolecules that contribute to aging. While there is currently no direct evidence that increased autophagy in older individuals can delay aging, it has been shown in C. elegans that autophagy is required for the lifespan extension of worms mutant for the insulin receptor *daf-2* [58]. In addition, a rodent study reporting the effects of every-other-day feeding on lifespan [41,43] is also compatible with an autophagy-based mechanism. In this experiment, rodents were dietarily restricted by allowing them to feed *ad libitum* during restricted hours. During the periods of feeding, the restricted cohort consumed almost the same quantity as rodents fed *ad libitum* all the time. These “restricted” animals were long-lived despite consuming almost the same diet as those fed *ad libitum* and achieved a very similar body weight. Thus, for this intervention at least, the advantage gained by the long-lived animals was not due to reduced calorie or food intake, but was acquired by periods of fasting—a concept remarkably similar to Kopeć's “antitoxins” furnished by periods of starvation [5], and compatible with an autophagy-like process acting to recycle cellular material to scavenge resources. Clearly, there is a need for further work to determine if an organism's response to DR is autophagy-dependent and if maintaining autophagy in an active state for longer can prolong lifespan.

## The Consequences of Sex during DR

One of the long-known features of DR animals is that they have compromised reproductive capacity. Indeed, this feature of DR has been used to form hypotheses about the evolutionary significance of the response to DR, based on the concept of allocation of limiting resources between somatic maintenance and reproduction [59–62]. For many organisms, it could be beneficial during lean times to increase allocation of nutritional resources into maintaining the adult, to increase the chance of survival to a time when the food supply is restored. In *Drosophila*, high levels of nutrition increase female egg production. This leads them to use up their supplies of stored sperm more rapidly [63] and to re-mate more frequently than those with poorer nourishment [17]. Sexual activity has been shown to shorten the lifespan of both male and female flies [14,64–66]. Thus, flies that are allowed to mate freely throughout life maintained on high-nutrient food will be shorter lived than those on low-nutrient food, due to differences in sexual activity, as well as differences caused by direct nutritional effects of DR. For this reason, it is essential for any study investigating the effects of DR on fly lifespan to control mating status; for instance, by using single-sex cohorts. At this stage it is unknown whether male–male or female–female interactions such as fighting [67] also affect lifespan in a nutrient-level-dependent manner. While the effect of adult density on lifespan is already known [68], the difference in longevity between individually and group-housed flies at different food levels remains untested.

Many studies that have used *Drosophila* to investigate the mechanisms of DR have done so using mixed-sex groups [20,26,50,69–77] (Table S1). From this body of work, it has been concluded that p53, SIR2, and resveratrol function in the same pathway, which is essential for DR's lifespan-extending effects [73–75]. However, the use of mixed-sex groups without controlling for mating status in these experiments means it is impossible to discern the interaction between genotype or drug treatment and diet on lifespan, because sexual activity will act as a confounding variable. Thus, it is important for future work on flies, as well as comparative mechanistic studies of DR that flies are housed in single-sex cohorts, or even singly, for experiments.

## Aging, Diseases, and Death

The long history of DR studies in rodents has provided us with a great deal of data that show that, with few exceptions, DR extends the lifespan of a variety of lab-maintained strains of rats and mice [7]. Many of these are inbred strains that are theoretically genetically homogenous and homozygous at almost all loci. Inevitably, different inbred lines become prone to different aging-related disorders [78], and yet they generally respond to DR with extended lifespan and a delay in a host of aging related diseases [7,39,40,79–81]. What is more, rodent models of specific diseases that are associated with aging, such as Alzheimer disease, appear to respond favourably to DR, with a delay in disease [82,83]. These observations and DR's ability to slow a wide array of aging-related disorders, as well as the evolutionary conservation of its lifespan-extending effect, can be interpreted to indicate that DR affects the root of aging itself and is not just reducing a specific disease by diet interaction [7]. If a similarly deep-rooted mechanism is at work in flies, a direct comparison of a variety of inbred laboratory lines of *Drosophila* should reveal that most respond to DR. This should also be true of flies affected by specific aging-related disorders, such as the fly model of Alzheimer disease [84]. As a by-product of this work, any mutants or inbred lines that do not respond to DR will be important tools in future work to uncover the mechanisms of lifespan extension.

## Conclusions

Despite many years of work, the mechanisms that underlie the effect of DR on lifespan remain unknown. Although historically much of the work has been performed with rodents, large-scale lifespan experiments under many conditions and genetic analysis are better suited to shorter-lived, and more easily housed, model organisms such as the invertebrates. However, if work in invertebrates is to be of any relevance to the study of aging in higher organisms, it is important to establish techniques that eliminate the confounding effects of nonaging-related causes of death, such as food toxicity and altered sexual activity. Only then can the mechanistic relationship between diet and death be established, providing modes of action to be tested in the longer-lived models. To test whether the mechanisms of DR are likely to be conserved from flies to mice, it will be interesting to see if *Drosophila* of different inbred lines or disease models respond to DR in a manner similar to their rodent counterparts. These studies are likely to be further refined by dietary interventions that focus on altering specific nutrients and on discovering mutations that block or alter the response to DR. What is exciting about studying DR in *Drosophila* is that each of the tools to perform these investigations are currently available or within close reach, meaning that characterisation of DR in *Drosophila* is likely to continue to be a fertile ground for research.

## Supporting Information

Table S1 Summary of Various DR Experiments Performed with Flies (96 KB DOC)

Video S1 Female *Drosophila* Feeding on DR and Control Media (9.2 MB WMV)

## Abbreviations

DR: dietary restriction
