# Supplementary material for: Dietary Restriction in Drosophila: Delayed Aging or Experimental Artefact?
Source: PLoS Genet. 2007 Apr 27;3(4):e57. doi: 10.1371/journal.pgen.0030057 (PMC1857724; doi:10.1371/journal.pgen.0030057)
Supplement: Table S1 — (96 KB DOC) [file pgen.0030057.st001.doc]

**Supplementary Table 1**

Summary of various DR experiments performed with flies.

| Intervention to restrict dietary intake | Diet | Mating status | Notes[[1]](#endnote-2) | Ref |
| --- | --- | --- | --- | --- |
|  |  |  |  |  |
| Dietary additions | Various | Various | These articles are sometimes cited as DR but largely study the effects of malnourishment | [1-8] |
|  |  |  |  |  |
| **No lifespan extension by food reduction - Intermittent feeding** | | | | |
|  |  |  |  |  |
| Intermittent daily starvation | Cornmeal, agar, malt extract | Single sex | - fecundity not applicable (males)  - experiments both with and without free access to water  - greatest longevity without starvation | [9] |
| Intermittent daily starvation | Cornmeal, sugar, agar, killed yeast, live yeast | Single sex | - female fecundity not reported  - free access to fresh water  - intermittent fasting not applied during weekends  - greatest longevity without starvation | [10] |
| Fixed quantity of limited food supplied daily | Dilutions of a Sugar / hydrolysed yeast solution | Single sex | - medflies  - reproduction increased with food availability  - no free access to water  - longest lifespan at greatest nutrient availability | [11] |
| Various quantities of limiting food supplied daily | Sucrose only or self-selected diet | Single sex | - house flies  - fecundity not applicable (males)  - longest lifespan at greatest nutrient availability | [12] |
|  |  |  |  |  |
| **No lifespan extension by food reduction – food dilution or nutrient manipulation** | | | | |
|  |  |  |  |  |
| Whole-food dilution | Dried yeast, cornflour, agar | Mixed sexes | - increased fecundity for each food increase  - lifespan increase to plateau at highest food concentrations | [13] |
| Dilution of added live yeast | S101 (sugar, minerals, salts, agar) from ref [14] | Mixed and Single sex cohorts | - female fecundity not reported, technique modified from [15]  - no lifespan difference between yeast dilution groups  - food quantity consumed per fly not determined before applying food dilution | [16] |
|  |  |  |  |  |
| **Lifespan extension reported for food reduction – food dilution or nutrient manipulation** | | | | |
|  |  |  |  |  |
| Isoenergetic replacement of carbohydrate and lipid components | various carbohydrate and fats in maize, rolled oats, dried yeast, agar | Single sex | - fecundity not possible (males)  - variety of effects on longevity  - without external health indicator it is impossible to know if flies can digest nutrient sources | [17-19] |
| Dilution of dietary protein | Sugar, agar and casein or dried yeast | Mixed sexes | - fecundity poor on all casein-based diets, but high with yeast addition  - lifespan greatest on yeast based diets (casein poorly nutritious)  - next longest lifespan on intermediate casein concentration | [20] |
| Manipulation of dietary components and pH | Semi-defined diets of vitamins, sucrose, casein (various pH) | Mixed sexes | - fecundity not determined  - peak lifespan on yeast-based diet than any defined condition (defined conditions poorly nutritious) | [21] |
| Yeast extract dilution | Cornmeal, yeast extract, sugar, agar | Single sex | - fecundity not relevant (males)  - lifespan decrease for each yeast extract increase. For controls peak at no yeast extract addition indicating toxicity  - DR response altered by genotype | [22] |
| Yeast component dilution | Cornmeal, dried yeast, sugar, agar | Mixed sexes | - fecundity not reported  - longest lifespan at intermediate yeast concentration | [23] |
| Addition of yeast | Sugar alone or sugar, yeast | Mixed sexes | - medflies  - increase in fecundity with yeast addition  - remaining life expectancy changed by yeast addition (increase then rapid decline) | [24] |
| Yeast and sugar components varied independently | Dried yeast, sugar, agar | Single sex | - fecundity not reported, food range from [25]  - yeast dilution accounted for majority of DR effect | [26] |
| Dilution of added live yeast solution | Sugar, charcoal, agar | Mixed sexes | - increased fecundity with increased yeast  - low yeast condition mostly longer-lived than controls (excl. control lines selected for desiccation resistance) | [15] |
| Diet change and dietary dilution | Dried yeast, sugar, cornmeal, agar or dried yeast, sugar, agar | Mixed sexes | - fecundity not reported, sugar/yeast food from [25]  - lifespan effect of DR effect blocked by mutation | [27] |
| Diet change | Dried yeast, sugar, cornmeal, agar or dried yeast, sugar, agar | Single sex | - fecundity not reported, sugar/yeast food from [25]  - two different food types used for ‘low’ and ‘high’ food  - lifespan effect of DR blocked by mutation | [28] |
| Dietary dilution | Cornmeal, yeast extract, sugar, agar | Single sex | - fecundity not determined  - lifespan peak at intermediate food concentration | [29] |
| Dietary dilution | Dried yeast, sugar, agar | Mixed sexes | - controlled mating status  - increasing fecundity with increasing food concentration  - demonstrated interaction between DR and sex | [25] |
| Dietary dilution | Dried yeast, sugar, agar | Single sex | - fecundity not reported, food range applied from [25] for same genetic stock  - lifespan longest at intermediate food concentration  - response to DR altered by genotype | [30] |
| Dietary dilution | Dried yeast, sugar, agar | Single sex | - fecundity not reported, food range applied from [25] for same genetic stock  - longest lifespan with intermediate food concentration | [31-37] |
| Dietary dilution | Dried yeast, sugar, agar | Mixed sexes | - fecundity not reported, food range from [25]  - response to DR altered by genotype | [38] |
| Dietary dilution | Dried yeast, sugar, agar | Mixed sexes | - fecundity for highest and lowest food concentrations reported  - lifespan peak at lowest food concentration | [39] |
| Dietary dilution | Dried yeast, sugar, agar | Mixed sexes | - fecundity not reported, food range from [39]  - lifespan peak at lowest food concentration | [40] |
| Dietary dilution and effect of live yeast olfaction and dilution | Dried yeast, sugar, agar | Single sex | - increased egg-laying with exposure to live yeast  - lifespan peak on intermediate food concentration, shortened by exposure to higher food and live-yeast or its odour alone | [41] |
| Dietary dilution | Dried yeast, sugar, agar | Single sex | - optimum sugar concentration found by monitoring egg-laying  - lifespan peak at intermediate yeast concentration  - found one yeast better than others for DR with *Drosophila* | [42] |

**References**

1. Northrop, JH (1917) The effect of prolongation of the period of growth on the total duration of life. J Biol Chem 32: 123-126.

2. Loeb, J, Northrop, JH (1917) On the influence of food and temperature upon the duration of life. J Biol Chem 32: 103-121.

3. Alpatov, WW (1930) Experimental Studies on the Duration of Life. XIII. The Influence of Different Feeding during the Larval and Imaginal Stages on the Duration of Life of the Imago of Drosophila melanogaster. American Naturalist 64: 37-55.

4. Hassett, CC (1948) The utilization of sugars and other substances by Drosophila. Biol Bull 95: 114-123.

5. Burcombe, JV, Hollingsworth, MJ (1970) The total nitrogen, protein, amino acid and uric acid content of ageing Drosophila. Exp Gerontol 5: 247-255.

6. Hollingsworth, MJ, Burcombe, JV (1970) The nutritional requirements for longevity in Drosophila. J Insect Physiol 16: 1017-1025.

7. Kircher, HW, Al Azawi, B (1985) Longevity of seven species of cactophilic Drosophila and D. melanogaster on carbohydrates. J Insect Physiol 31: 165-169.

8. Good, TP, Tatar, M (2001) Age-specific mortality and reproduction respond to adult dietary restriction in Drosophila melanogaster. J Insect Physiol 47: 1467-1473.

9. Kopec, S (1928) On the influence of intermittent starvation on the longevity of the imaginal stage of *Drosophila melanogaster*. British J Exp Biol 5: 204-211.

10. Le Bourg, E, Medioni, J (1991) Food Restriction and Longevity in *Drosophila melanogaster*. Age & Nutrition 2: 90-94.

11. Carey, JR, Liedo, P, Harshman, L, Zhang, Y, Muller, HG et al. (2002) Life history response of Mediterranean fruit flies to dietary restriction. Aging Cell 1: 140-148.

12. Cooper, TM, Mockett, RJ, Sohal, BH, Sohal, RS, Orr, WC (2004) Effect of caloric restriction on life span of the housefly, Musca domestica. FASEB J 18: 1591-1593.

13. David, J, van Herrewege, J, Fouillet, P (1971) Quantitative under-feeding of Drosophila: effects on adult longevity and fecundity. Exp Gerontol 6: 249-257.

14. Pearl, R, Allen, A, Penniman, WBD (1926) Culture Media for Drosophila. II. A New Synthetic Medium and Its Influence on Fertility at Different Densities of Population. American Naturalist 60: 357-366.

15. Chippindale, AK, Leroi, AM, Kim, SB, Rose, MR (1993) Phenotypic plasticity and selection in Drosophila life-history evolution. I. Nutrition and the cost of reproduction. J Evol Biol 6: 171-193.

16. Le Bourg, E, Minois, N (1996) Failure to confirm increased longevity in Drosophila melanogaster submitted to a food restriction procedure. J Gerontol A Biol Sci Med Sci 51: B280-B283.

17. Driver, CJ, Cosopodiotis, G (1979) The effect of dietary fat on longevity of Drosophila melanogaster. Exp Gerontol 14: 95-100.

18. Driver, CJ, Lamb, MJ (1980) Metabolic changes in ageing Drosophila melanogaster. Exp Gerontol 15: 167-175.

19. Driver, CJ, Wallis, R, Cosopodiotis, G, Ettershank, G (1986) Is a fat metabolite the major diet dependent accelerator of aging? Exp Gerontol 21: 497-507.

20. Min, KJ, Tatar, M (2006) Restriction of amino acids extends lifespan in Drosophila melanogaster. Mech Ageing Dev 127: 643-646.

21. Van Herrewege, J (1974) Nutritional requirements of adult Drosophila melanogaster: the influence of the casein concentration on the duration of life. Exp Gerontol 9: 191-198.

22. Kapahi, P, Zid, BM, Harper, T, Koslover, D, Sapin, V et al. (2004) Regulation of Lifespan in Drosophila by Modulation of Genes in the TOR Signaling Pathway. Curr Biol 14: 885-890.

23. Min, KJ, Tatar, M (2006) Drosophila diet restriction in practice: Do flies consume fewer nutrients? Mech Ageing Dev 127: 93-96.

24. Carey, JR, Liedo, P, Muller, HG, Wang, JL, Vaupel, JW (1998) Dual modes of aging in Mediterranean fruit fly females. Science 281: 996-998.

25. Chapman, T, Partridge, L (1996) Female fitness in Drosophila melanogaster: an interaction between the effect of nutrition and of encounter rate with males. Proc R Soc Lond B Biol Sci 263: 755-759.

26. Mair, W, Piper, MD, Partridge, L (2005) Calories do not explain extension of lifespan by dietary restriction in *Drosophila*. Public Library of Science: Biology 7: e223-

27. Rogina, B, Helfand, SL (2004) Sir2 mediates longevity in the fly through a pathway related to calorie restriction. Proc Natl Acad Sci U S A 101: 12980-12985.

28. Rogina, B, Helfand, SL, Frankel, S (2002) Longevity regulation by Drosophila Rpd3 deacetylase and caloric restriction. Science 298: 1745-

29. Carvalho, GB, Kapahi, P, Benzer, S (2005) Compensatory ingestion upon dietary restriction in Drosophila melanogaster. Nat Methods 2: 813-815.

30. Clancy, DJ, Gems, D, Hafen, E, Leevers, SJ, Partridge, L (2002) Dietary restriction in long-lived dwarf flies. Science 296: 319-

31. Pletcher, SD, Macdonald, SJ, Marguerie, R, Certa, U, Stearns, SC et al. (2002) Genome-wide transcript profiles in aging and calorically restricted Drosophila melanogaster. Curr Biol 12: 712-723.

32. Mair, W, Goymer, P, Pletcher, SD, Partridge, L (2003) Demography of Dietary Restriction and Death in Drosophila. Science 301: 1731-1733.

33. Miwa, S, Riyahi, K, Partridge, L, Brand, MD (2004) Lack of correlation between mitochondrial reactive oxygen species production and life span in Drosophila. Ann N Y Acad Sci 1019:388-91.: 388-391.

34. Magwere, T, Chapman, T, Partridge, L (2004) Sex differences in the effect of dietary restriction on life span and mortality rates in female and male Drosophila melanogaster. J Gerontol A Biol Sci Med Sci 59: 3-9.

35. Mair, W, Sgro, CM, Johnson, AP, Chapman, T, Partridge, L (2004) Lifespan extension by dietary restriction in female Drosophila melanogaster is not caused by a reduction in vitellogenesis or ovarian activity. Experimental Gerontology 39: 1011-1019.

36. Hulbert, AJ, Clancy, DJ, Mair, W, Braeckman, BP, Gems, D et al. (2004) Metabolic rate is not reduced by dietary-restriction or by lowered insulin/IGF-1 signalling and is not correlated with individual lifespan in Drosophila melanogaster. Exp Gerontol 39: 1137-1143.

37. Magwere, T, Goodall, S, Skepper, J, Mair, W, Brand, MD et al. (2006) The effect of dietary restriction on mitochondrial protein density and flight muscle mitochondrial morphology in Drosophila. J Gerontol A Biol Sci Med Sci 61: 36-47.

38. Bauer, JH, Poon, PC, Glatt-Deeley, H, Abrams, JM, Helfand, SL (2005) Neuronal expression of p53 dominant-negative proteins in adult Drosophila melanogaster extends life span. Curr Biol 15: 2063-2068.

39. Bross, TG, Rogina, B, Helfand, SL (2005) Behavioral, physical, and demographic changes in Drosophila populations through dietary restriction. Aging Cell 4: 309-317.

40. Zheng, J, Mutcherson, R, Helfand, SL (2005) Calorie restriction delays lipid oxidative damage in Drosophila melanogaster. Aging Cell 4: 209-216.

41. Libert, S, Zwiener, J, Chu, X, Vanvoorhies, W, Roman, G et al. (2007) Regulation of Drosophila life span by olfaction and food-derived odors. Science 315: 1133-1137.

42. Bass TM, Piper MD, Grandison RC, Wong R, Martinez P et al. (2007) Optimization of dietary resitrction protocols for *Drosophila* to avoid food toxicity. J Gerontol. In Press.

1. Except where indicated, studies were performed with *Drosophila* *melanogaster* [↑](#endnote-ref-2)
